# Comparative Analysis of the Mitochondrial Genomes of *Nicotiana tabacum*: Hints Toward the Key Factors Closely Related to the Cytoplasmic Male Sterility Mechanism

**DOI:** 10.3389/fgene.2020.00257

**Published:** 2020-03-20

**Authors:** Ruyi Wang, Xunhui Cai, Shengnan Hu, Ying Li, Yanjun Fan, Siqiao Tan, Qiyuan Liu, Wei Zhou

**Affiliations:** ^1^Hunan Engineering and Technology Research Center for Agricultural Big Data Analysis and Decision-Making, Changsha, China; ^2^Hunan Provincial Key Laboratory for Biology and Control of Plant Diseases and Insect Pests, College of Plant Protection, Hunan Agricultural University, Changsha, China; ^3^School of Electronic Information and Communications, Huazhong University of Science and Technology, Wuhan, China; ^4^Hunan Engineer Research Center for Information Technology in Agriculture, Changsha, China; ^5^Key Laboratory of Crop Physiology, Ecology and Genetic Breeding, Ministry of Education, College of Agronomy, Jiangxi Agricultural University, Nanchang, China

**Keywords:** *Nicotiana tabacum*, mitochondrial genome, comparative analysis, cytoplasmic male sterility, rearrangement

## Abstract

**Background:**

Cytoplasmic male sterility (CMS) is a complex phenomenon of plant sterility that can produce non-functional pollen. It is caused by mutation, rearrangement or recombination in the mitochondrial genome. So far, the systematic structural characteristics of the changes in the mitochondrial genome from the maintainer lines to the CMS lines have not been reported in tobacco.

**Results:**

The mitochondrial genomes of the flower buds from both CMS lines and maintainer lines of two *Nicotiana tabacum* cultivars (YY85, sYY85, ZY90, and sZY90) were sequenced using the PacBio and Illumina Hiseq technology, and several findings were produced by comparative analysis based on the *de novo* sequencing. (1) The genomes of the CMS lines were larger, and the different areas were mostly non-coding regions. (2) A large number of rearrangement regions were detected in the CMS lines, with many translocation regions. (3) Thirteen gene clusters were shared by the four mitochondrial genomes, among which two of the gene clusters, *nad2-sdh3* and *nad6-rps4*, were far from each other in the CMS lines. (4) Thirty-three protein-coding genes were conserved in four mitochondrial genomes. However, *nad3* was detected one additional copy in the maintainer lines, and sequence differences were revealed in the four candidate genes (*atp6*, *cox2*, *nad2*, and *sdh3*). Importantly, the evolutionary tree based on the four genes could be used to distinguish the CMS lines and the maintainer lines well for the sequenced mitochondrial genomes of the tobacco. (5) Sixteen CMS-specific open reading frames (ORFs) were found, three of which (*orf91*, *orf115b*, and *orf100*) were previously reported. (6) The differences in intensity of the protein–protein (PPI) interaction in ATP6 were further verified using the yeast two-hybrid analysis.

**Conclusion:**

Although the majority of the sequences, genes and gene clusters were shared by the mitochondrial genomes of the maintainer and the CMS lines in tobacco, extensive structural variations identified with comprehensive analysis based on the mitochondrial genomes, including rearrangement, gene order, the mitochondrial genome expansion and shrinkage events, might be related to CMS. Additionally, the candidate protein-coding genes and CMS-specific ORFs were closely associated with the CMS mechanism. Verification experiments of one of the candidate genes were performed, and the validity of our research results was supported.

## Introduction

As of September 2019, a total of 10,249 mitochondrial genomes, including 9,034, 616, 163, 290 and 146 mitochondrial genomes of animals, fungi, protists, plants, and others, respectively, were collected in the NCBI. The plant mitochondrial genome is highly variable: the smallest mitochondrial genome that has been sequenced is from *Brassica napus* with only 221 kb (Handa, 2003), and the largest one is from *Cucumis melo* with 2,740 kb (Rodriguez-Moreno et al., 2011). Tobacco belongs to Solanaceae, which also includes the potato, tomato, henbane, petunia, eggplant, pepper, etc. The mitochondrial genome size of Solanaceae plants varies from 390 to 530 kb, and the number of coding genes is similar among these plants (Nivison et al., 1994; Xu et al., 2011; Hirakawa et al., 2014; Jo et al., 2014; Sanchez-Puerta et al., 2015). At present, the mitochondrial genome data of Solanaceae are mostly next generation sequencing data, but third generation sequencing data is lacking. The structure of plant mitochondrial genome is very complex and requires third generation sequencing technology, and third generation sequencing technology is widely used in methylation research, mutation identification and detection of DNA modifications (Xiao et al., 2017, 2018; Liu et al., 2019). The majority of plant mitochondrial genomes are non-coding sequences composed of repeated sequences, gene spacing sequences and introns. Furthermore, there are many homologous DNA sequences in the plant mitochondrial genomes, which make the plant mitochondrial genomes larger and more complex than those of animals. In addition, the mitochondrial genome in most plants has many subgenomic molecules (also called sblimons) (Li et al., 2017).

The molecular mechanism of cytoplasmic male sterility (CMS) in plants is very complex and comes from a wide range of sources, and the plant mitochondrial genome usually serves as the carrier of CMS. CMS phenomena may be caused by events that affect the structure of the mitochondrial genome or mitochondrial gene function in plants. CMS has been identified in over 150 species (Schnable and Wise, 1998). CMS is widely used in hybrid seed production, which eliminates the need to remove stamens manually, saving time and labor. Since 1970, a series of CMS molecular mechanism studies have been performed in maize, petunia, sorghum, rice, *Brassica napus*, wheat, sugar beet, sunflower, radish and other crops (Pring and Levings, 1978; Ogihara et al., 2005; Chen and Liu, 2014). Li et al. (1988) provided the first evidence that mitochondrial gene mutation could cause CMS or CMS genes to be mitochondrial genes. They also found that variations in the mitochondrial genome, stoichiometric variations in mitochondrial subgenomic molecules and the expression of specific mitochondrial gene sequences had impacts on CMS (Li et al., 1988; Hanson, 1991; Budar et al., 2003; Arrieta-Montiel and Mackenzie, 2011). It is indicated that CMS is closely related to variations in the mitochondrial genome, and CMS genes are located in the mitochondrial genome. Changes in the plant mitochondrial genome structure can be caused by homologous recombination events, and repeat sequences play an important role. The number of repeated sequences can be altered by events mediated by repeated sequences of genome rearrangement and variations in the mitochondrial genome structure may be caused under certain conditions. Therefore, the structure and evolution of the plant mitochondrial genomes have been greatly affected by repeated sequences (Bellaoui et al., 1998; Kanazawa et al., 1998; Satoh et al., 2006).

Besides rearrangement of the mitochondrial genome, plant CMS can also be caused by the specific expression of CMS-associated genes in the mitochondrial genome. CMS-associated genes appear to be the result of recombination events that produce new open reading frames (ORFs), usually recombination between mitochondrial genes and unknown ORF or coding regions. Moreover, CMS-associated genes are usually located near or are parts of an *atp* gene and are co-transcribed with the flanking mitochondrial gene (Hanson and Bentolila, 2004). Many CMS-associated genes have similar expression profiles to the standard mitochondrial gene, with which they are co-transcribed. Furthermore, they are expressed throughout the plant (Kempken, 2011). CMS-associated genes have been identified in many plants. However, CMS-associated genes are not the same in the sterile lines of different plants. In rice, *orf79* for CMS-BT derived from the *indica* rice variety Boro II, and its variant *orfH79* for CMS-HL derived from the wild rice accession Hong-Lian, encoded proteins with a 5′ region similar to *cox1* and a 3′ region of unknown origin (Wang et al., 2006; Peng et al., 2010). In sorghum CMS-A3, the chimeric *orf107* had a similar segment to *atp9* and a remaining portion that was similar to *orf79* (Tang et al., 1996). In a chimeric wheat ORF associated with CMS, the first 11 amino acids encoded by *orf256* were the same as the first 11 amino acids encoded by *cox1*. The *orf256* gene region was apparently a combination of 261 bp of the *cox1* gene region, including 228 bp from the 5′ flanking region, 33 bp from the encoded N-terminus and a segment of DNA from an unknown origin (Hedgcoth et al., 2002). In *Brassica*, *orf222* of CMS-nap, which potentially encoded a protein with 222 amino acids, possessed 79% sequence similarity to the predicted product of *orf224* of CMS-pol (Singh and Brown, 1991; L’homme et al., 1997). Approximately 60 amino acids at the extreme N-terminus of the encoded protein were derived from a normal mitochondrial gene, namely *atp8* (or *orfB*) (Geddy et al., 2005). The *Brassica* CMS-tour gene *orf263* was found to be highly similar (94%) to the 5′ part of the *nad5a* and highly homologous (92%) to the *atp9* gene (Landgren et al., 1996). In sunflower CMS-Baso, the CMS-associated gene *orfH522* had a homologous region with *orfB* and *atp8* (Kohler et al., 1991). In petunia, a gene fusion (the *Pcf* gene) was found to contain the 5′-flanking and amino-terminal transmembrane segment of *atp9*, parts of the *cox2* coding region, and the carboxyl terminus and 3′-flanking region of an unidentified reading frame *urfS* (Young and Hanson, 1987).

In order to research the CMS mechanism more systematically, four mitochondrial genomes of both CMS lines and maintainer lines of two *Nicotiana tabacum* cultivars were sequenced in this study. Comparative analysis between the normal and CMS mitochondrial genomes will help with the study of the CMS mechanism and the screening of CMS-associated genes in plants.

## Materials and Methods

### Plant Materials

Both CMS lines and maintainer lines of two *Nicotiana tabacum* cultivars were used in our study: (1) the maintainer line (YY85, GenBank: MN651321) and its CMS line (sYY85, GenBank: MN651322) of yunyan85, (2) the maintainer line (ZY90, GenBank: MN651323) and its CMS line (sZY90, GenBank: MN651324) of zhongyan90. The cytoplasm of sYY85 and sZY90 was derived from the sun-cured tobacco cultivar “tiegu” of Jiangxi province, China. All cultivars were bred successively for more than 10 consecutive generations so that their CMS lines were stable, and their nuclear backgrounds were the same as that of the maintainer lines.

### Mitochondrial DNA Extraction and DNA Library Construction

mtDNA was isolated from approximately 5 g of fresh leaves using an improved extraction method (Chen et al., 2011). According to the manufacturer’s instructions, mtDNA was extracted using the E.Z.N.A^®^ Plant kit (OMEGA). After DNA extracting, 1 μg of purified DNA was fragmented to construct the short-insert mitochondrial genome libraries (insert size 430 bp) using the TruSeq^TM^ Nano DNA Sample Prep Kit (Illumina), which was then sequenced on the Illumina Hiseq 4000 (Borgstrom et al., 2011). The high molecular weight DNA was purified and used for PacBio library (8–10 kb) preparation, subjected to BluePippin size selection and then sequenced on the Sequel Sequencer.

### DNA Sequencing

Illumina Hiseq and PacBio sequencing technologies were used to sequence the mitochondrial genome, respectively. The original Illumina Hiseq sequence image data were converted to sequence data by base calling, and the results were stored in the FASTQ file format. The FASTQ file was the most original data file, and it contained sequencing read information and sequencing quality information. The Illumina Hiseq sequencing platform was used to sequence four samples, but a certain proportion of raw data was of low quality. To make the subsequent analysis results more accurate and reliable, the original sequencing data were processed as follows: (1) the adapter sequences of reads were removed; (2) non-AGCT bases at the 5′ end were removed before shearing; (3) read ends with low sequencing quality (Q < 20) were filtered; (4) reads that contained uncalled bases (“N” characters) greater than a ratio of 10% were removed; (5) adapter and quality trimmed short segments less than 50 bp in length were discarded. PacBio Sequel sequenced data were stored in the bam format and could be converted to FASTA or FASTQ format. The raw data of the PacBio Sequel platform had some defects such as connector sequences, low-quality sequences and sequencing errors. To obtain more accurate assembly results, the original sequencing data were processed as follows: (1) polymerase reads with length less than 100 bp were filtered; (2) polymerase reads of quality less than 0.80 were removed; (3) subreads from the polymerase reads were extracted and the adapter sequences were filtered; (4) subreads with length less than 500 bp were removed.

### Genome Assembly

The mitochondrial genome was assembled using a combination of the PacBio Sequel and the Illumina Hiseq data using the following steps. Firstly, the genome framework for Illumina and PacBio data was assembled using SPAdes v3.10.1 (Antipov et al., 2016). The specific process was as follows: (1) Illumina sequencing data with SOAPdenovo v2.04 were assemble preliminarily, and then PacBio sequencing data were aligned with BLASR (Basic Local Alignment with Successive Refinement); (2) the calibration was carried out to reduce the single-molecule long sequence of the bases and insert the missing errors (the error caused by the insertion and deletion in the single-molecule long sequence of the bases) according to the alignment results of single-molecule sequencing data; (3) the corrected PacBio Sequel data were mixed with the Illumina Hiseq data and then SPAdes v3.10.1 (Antipov et al., 2016) were used to assemble; (4) sequences with sufficient coverage depth and long assembly length were selected as candidate sequences and compared with the NT library for confirmation; (5) Illumina data were used to verify the final assembly result. Secondly, the assembly was verified and the circle or linear mitochondrial genome was completed by filling in any gaps. Finally, clean reads were mapped to the assembled mitochondrial genome to correct the wrong bases and determine if any insertions and deletions were present (Koren et al., 2012).

### Repeated Sequences Analysis

The target mitochondrial genome sequences were aligned using the BLASTN algorithm^1^. BLASTN was used to obtain a library of repeated sequencesin which a given target sequence was both a query and a subject. A pair of sequences was defined as a repeat if it had more than 90% identity. According to the size of the repeated sequences, small (<50 bp), intermediate (50–500 bp) and large (>500 bp) repeats were defined (Yang et al., 2016). Simple sequence repeats (SSRs) or short tandem repeats (STRs) were found by Repeatmasker^2^.

### Syntenic Analysis of the CMS Lines and the Maintainer Lines

The synteny of the mitochondrial genome referred to the phenomenon that homologous genes and sequences of the different mitochondrial genomes were arranged in the same order. The extent of synteny between two mitochondrial genomes could be used to measure the evolutionary distance and the genetic relationship between the species. Using MUMmer, both CMS lines and maintainer lines of two *Nicotiana tabacum* cultivars were compared, and the wide homologous regions of them were determined. Translocation/Trans, Inversion/Inv, and Translocation and Inversion (Trans + Inv) regions were found using LASTZ v1.03.54.

### Gene Annotation and ORF Selection

Mitochondrial gene were annotated by homology alignments and *de novo* prediction, and EVidenceModeler v1.1.1 (Haas et al., 2008) was used to integrate the gene set. The protein sequences were quickly compared with the sample genome sequences, and low score alignment results were filtered to eliminate redundancy. The exact alignment was obtained from Genewise and by performing *de novo* gene prediction using AUGUSTUS^3^. Transfer RNA (tRNA) and ribosome RNA (rRNA) genes were predicted by tRNAscan-SE (Lowe and Eddy, 1997) and rRNAmmer (Lagesen et al., 2007), respectively. The circular mitochondrial genome was mapped by OrganellarGenomeDRAW v1.2 (Lohse et al., 2007). Potential ORFs > 300 bp were predicted using ORF-finder, blastn and blastx. The common ORFs that only existed in the CMS lines were identified using Matlab scripts.

### Protein–Protein Interaction (PPI) of One of the Candidate Genes

The protein–protein interaction (PPI) relationship of candidate genes was predicted by STRING9.1 and validated by the yeast two-hybrid experiment.

### Phylogenetic Analysis

All tobacco mitochondrial genomes in NCBI and the four mitochondrial genomes we sequenced were collected to construct two kinds of phylogenetic trees: (1) differential gene sequences based on ClustalW and MEGA v7.0 (using the Maximum Parsimony method and 1000 bootstrap replications); (2) a single nucleotide polymorphism (SNP) matrix based on the sample and reference genome (Bright Yellow 4, GenBank: BA000042). All SNPs were connected in the same order for each sample. PhyML v3.0 (using maximum likelihood method) was used to construct a phylogenetic tree with input files in fasta format and sequences of the same length.

## Results and Discussion

### Comparative Analysis of Features and Gene Contents in the Mitochondrial Genomes

The mitochondrial genome features were compared among YY85, sYY85, ZY90, and sZY90. The genomes of the CMS lines were significantly larger than those of their corresponding maintainer lines, which were expanded by about 30–60 kb (Table 1). The mutation rate measured in protein-coding regions and rRNA regions was very low. However, non-coding DNA evolved so rapidly that the plant mitochondrial genomes underwent major rearrangements and expansions (Palmer and Herbon, 1988; Christensen, 2013). Christensen suggested that the mitochondrial genomes were repaired differently in genes and junk regions. Double-strand break (DSB) repair might occur in either coding DNA or non-coding DNA, both of which were accurate and inaccurate, respectively. Most of the DNA repair was mediated via the generation of DSB and the inaccurate repair performance in non-coding sequences tended to introduce non-homologous sequences (Christensen, 2014). This could explain the evolution and expansion of the plant mitochondrial genomes. mtDNA expansion also occurred in the maintainer line (ZY100, GenBank: KR780036) and its CMS line (sZY100, GenBank: KR071121) of zhongyan100 from NCBI. In this case, the CMS mitochondrial genome was 90 kb larger than the ZY100 mitochondrial genome, which might be due to the inaccurate DSB repair of non-coding DNA, leading to the mitochondrial genome expansion. The GC proportion and the protein-coding gene number were similar among the four mitochondrial genomes, but the length of repeat sequences between the CMS lines and the maintainer lines was altered significantly (Table 1). The different mechanism of DSB could explain why DSB repair of non-coding sequences always led to genome recombination and repeated sequence changes (Kempken, 2011). The recombination of the plant mitochondrial genomes relied on the invasion of the DNA strand and DSB. In addition, the chloroplast-derived sequences of the four mitochondrial genomes were very similar and stable in both CMS lines and maintainer lines of two *Nicotiana tabacum* cultivars.

**TABLE 1:** Features of the mitochondrial genomes of the YY85, sYY85, ZY90, and sZY90 lines.

Feature	YY85	sYY85	ZY90	sZY90
Genome size (bp)	430,974	468,288	472,218	530,869
GC content (%)	39.76	40.05	39.33	40
Protein-coding genes (number)^a^	35	33	36	35
Coding sequence (bp)^a^	30,630	29,637	33,954	31,974
Coding sequence/genome (%)	7.11	6.33	7.19	6.02
Repeated sequence (bp)^b^	57,929	43,190	53,282	115,387
Repeated sequence/genome (%)	13.4	9.2	11.3	21.7
Chloroplast-derived sequence (%)^c^	2.6	2.4	2.8	2.1
Gene content (number)	62	61	65	68
rRNAs (number)	4	4	4	5
tRNAs (number)	23	24	25	28

*^*a*^Copies of duplicated genes were included.^*b*^Copies of each repeat were not considered. If the repeated sequence units overlapped, the overlapping sequence repetitions were calculated.^*c*^Copies of alignments were excluded. The chloroplast genomes (GenBank: Z00044.2) were aligned by BLASTN algorithm.*

**FIGURE 1:**
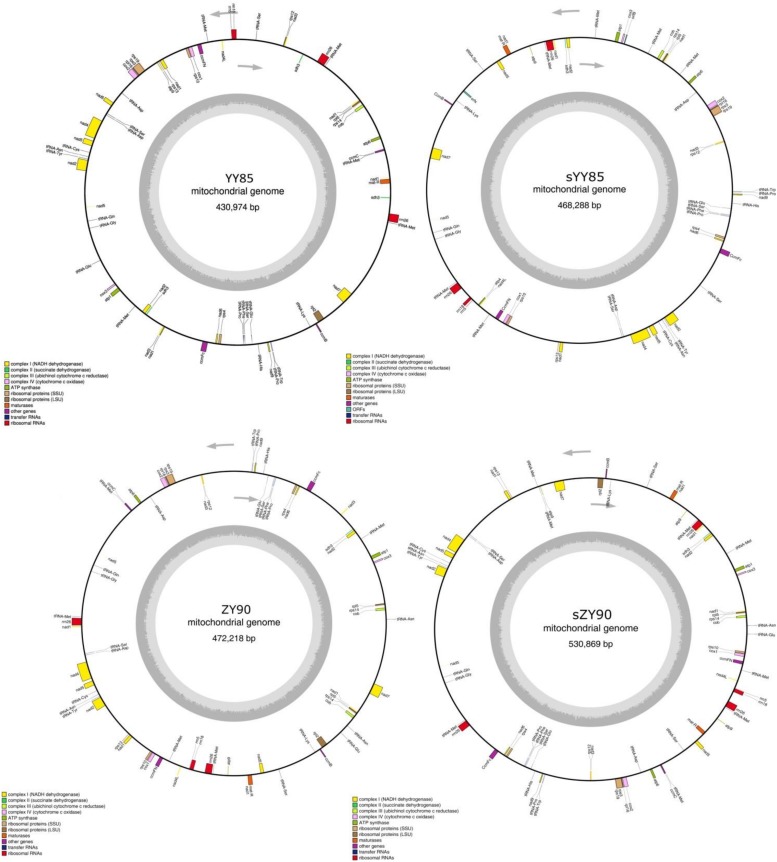
The four circular mitochondrial genomes of both CMS lines and maintainer lines of two *Nicotiana tabacum* cultivars. Gene names were shown outside the circle. The GC contents were shown inside the circle.

A total of 30 conserved coding genes were found among the four mitochondrial genomes (Table 2). Copies of *atp9*, *nad3*, *sdh3*, *cob*, *rpl5*, *rps14* and *mat-R*, as well as the existence of *ccmC*, *rpl2* and *atp4*, showed the differences in the conserved coding genes (Figure 1). Notably, there was another copy of *nad3* (*nad3-D2*) in both YY85 and ZY90. A reduced copy number of *nad3* might result in decreased expression of the gene in sYY85 and sZY90, which might further reduce mitochondrial energy supply and non-functional pollen production.

**TABLE 2:** The conserved genes of the YY85, sYY85, ZY90, and sZY90 lines.

Gene products	Gene names
ATP synthase	atp1; atp6; atp9* (1/1/1/3)
Cytochrome c biogenesis	*ccmB; ccmFC; ccmFN*
NADH dehydrogenase (complex I)	nad1; nad2; nad3* (2/1/2/1)*; nad4; nad4L; nad5; nad6; nad7; nad9*
Succinate dehydrogenase (complex II)	sdh3* (3/1/1/1)
Cytochrome b (complex III)	cob* (1/1/2/1)
Cytochrome c oxidase (complex IV)	*cox1; cox2; cox3*
Ribosomal proteins	rpl16; rpl5* (1/1/2/1); rps3; rps4; rps10; rps12; rps13; rps14* (1/1/2/1)*; rps19*
Maturases	mat-R* (1/1/1/2)

*These conserved genes have different copies in YY85, sYY85, ZY90, and sZY90.

### Repeated Sequence Comparison in the Mitochondrial Genomes

**FIGURE 2:**
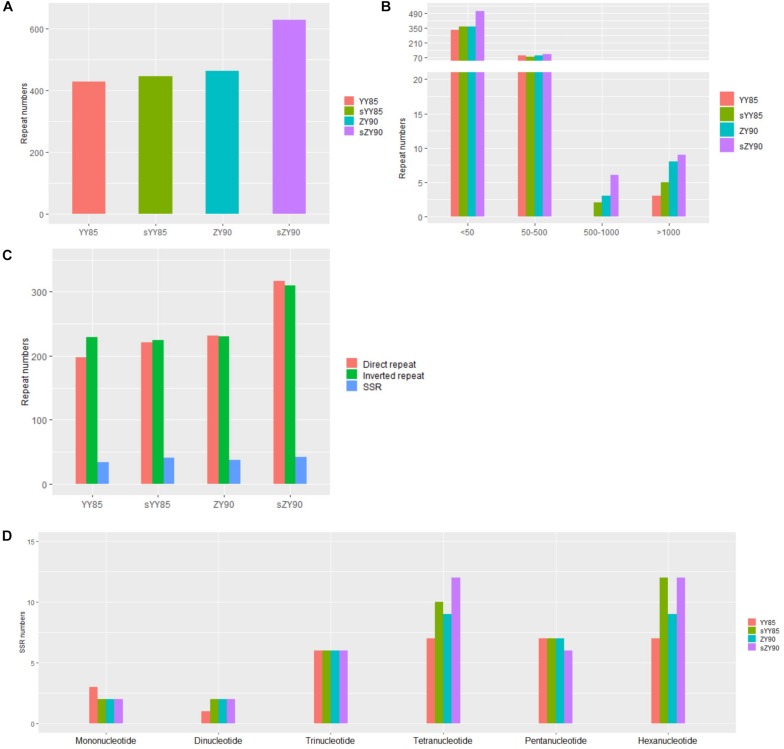
**(A)** Number of repeated sequences. **(B)** Number of repeated sequences by length. **(C)** Number of repeated sequences by type. **(D)** Number of SSRs by motifs sizes.

Repeated sequences were classified into large sequences (>500 bp), intermediate sequences (50–500 bp) and small sequences (<50 bp) (Figure 2B) according to their sizes. After identifying repeated sequences with BLAST, they were obviously that the lengths of repeated sequences changed significantly in the CMS lines (Table 1). It was found that in the sYY85 mitochondrial genome, there were 4 more large repeated sequences, 15 fewer intermediate ones and 30 more small ones. In the sZY90 mitochondrial genomes, 4 more large repeated sequences, 13 more intermediate ones and 148 more small ones were also found (Figure 2B). Meanwhile, the proportion of repeated sequences decreased by 4.2% in sYY85 and increased by 10.4% in sZY90 (Table 1). These changes might have been caused by mitochondrial DSB, which was a necessary condition for the recombination of the plant mitochondrial genome.

According to the arrangement direction, the repeated sequences were divided into direct repeat (DR) and inverted repeat (IR). A palindromic sequence is a kind of IR, in which the smaller palindromic sequence might be the cutting site of the restriction enzyme, while the larger palindromic sequence might be closely related to transcription termination. The number of IRs and DRs were similar and significantly higher than SSRs (Figure 2C). The total repeated sequence number concerned IRs, DRs and SSRs, and the number of repeated sequences in the CMS lines was greater than that of the maintainer lines (Figure 2A). The large repeated sequences of YY85 and sYY85 were then aligned, and so were those of ZY90 and sZY90. The results showed that 95% of the large repeated sequences in YY85 and 40% of those in sYY85 came from the homologous sequence region, as did 90% of the large repeated sequences in ZY90 and 55% of those in sZY90. Moreover, all of the large repeated sequences in YY85 and ZY90 were derived from the tobacco nuclear genome, as were 41% of the large repeated sequences in sYY85 and 58% of those in sZY90. This finding indicated that the increase in the number of large repeated sequences and the proportion of large repeated non-homologous sequences might represent another indicator of the transformation process from the tobacco maintainer to the CMS lines.

Five pairs of repeated sequences larger than 1 kb, namely 2.5, 2.9, 5.8, 4.2, and 8.1 kb were identified in the sYY85 mitochondrial genome; nine pairs, namely 1.5, 1.6 (2), 2.8, 4.3, 8.0, 8.5, 10.4 and 47.2 kb, were also identified in the sZY90 mitochondrial genome. These regions had high DNA exchange activity and provided some indication of possible hotspots for DSB event. It was likely that these repeated sequences played a role as replication origins in strand invasion-mediated replication initiation within large, linear DNA molecules (Zaegel et al., 2006). In sYY85 and sZY90, there was a pair of continuous inverted repeats that belonged to a pair of palindrome sequences without protein-coding genes, with sequences lengths of 5,778 bp (R5778) and 1,491 bp (R1491), respectively, but there were no similar findings in their maintainer lines. Large palindromic sequences were more likely to convert into hairpin structures to control the initiation of transcription. The gene *rps13* from sYY85 was located 14,350 bp downstream of R5778, and *rps13* from sZY90 was located 14,404 bp downstream of R1491. During transcription, repeats might be more likely to complement each other when double-stranded DNA is opened, forming a hairpin structure that terminates the transcription. This could affect the number of *rps13* transcripts and reduce their expression in mitochondria, thus disabling the already weak mitochondrial energy supply.

According to the length of tandem repeats, SSRs with motif sizes of 1–6 bp were detected (Figure 2D). We identified 34, 42, 37 and 42 SSRs in YY85, sYY85, ZY90, and sZY90, respectively, nearly 70% of which belonged to the tetramers (24.7%), the pentamers (17.5%) and the hexamers (26%), while the occurrence of frequency of the monomers, the dimers and the trimers was relatively low. The specific size and location of the SSRs are shown in Supplementary Table S1. The pentamers were conserved in the four mitochondrial genomes, whereas the tetramers and the hexamers were different in the maintainer and the CMS lines, where (CTTA)n and (CTCCAA)n were only derived from sYY85 and sZY90, respectively.

### Rearrangements of the Mitochondrial Genome Structure in the Maintainer Lines and the CMS Lines

**FIGURE 3:**
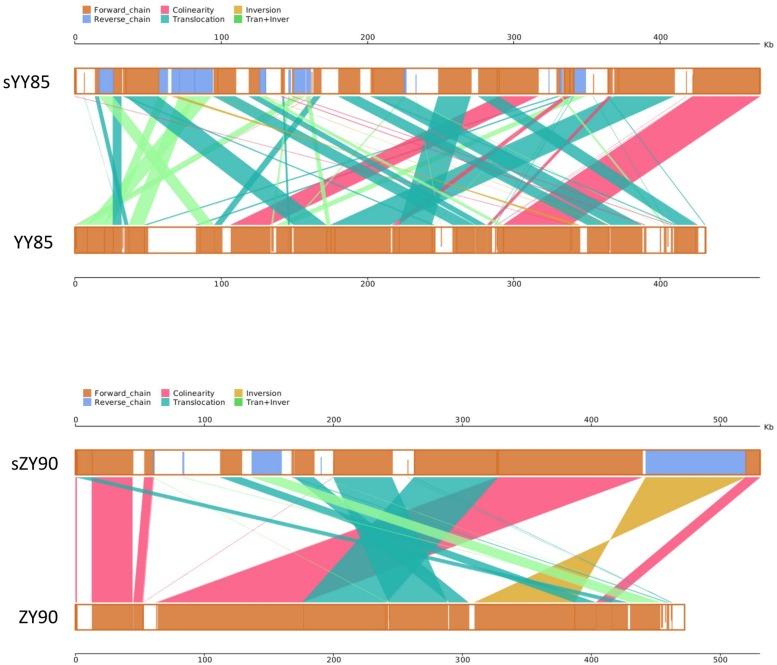
Parallel synteny graphs of the tobacco mitochondrial genomes. The yellow boxes in both panels represented the forward chain of the mitochondrial genome, and the blue boxed represented the reverse chain. The color depth in the boxes indicated similarity, and the full fill indicated 100% similarity. The color of connecting regions between the upper and lower axes indicated the alignment types: collinearity, translocation, inversion, translocation, and inversion regions.

It was found that 71.15% sequences of sYY85 could be aligned with 77.33% sequences of YY85, and 78.99% sequences of sZY90 could be aligned with 88% sequences of ZY90. Based on the homologous sequences and syntenic analysis of the four mitochondrial genomes, 45, 52, 25, and 27 homologous sequence regions (syntenic blocks) were found in YY85, sYY85, ZY90 and sZY90, respectively. In the syntenic region of YY85/sYY85 and ZY90/sZY90, there were 11 and 9 collinearity regions, 24 and 13 translocation regions, 3 and 1 inversion regions, and 14 and 4 simultaneous inversion and translocation regions (Supplementary Table S2). The rearrangement of the mitochondrial genome refers to the process in which genes and sequences are rearranged in the genome to obtain new genomes through evolution. These events mainly include inversion and translocation of sequences. In both the CMS lines and maintainer lines of two *Nicotiana tabacum* cultivars, more than 60% of the syntenic blocks were rearranged, and there were marked differences in the syntenic block order among the mitochondrial genomes. The rearrangements of the CMS mitochondrial genomes are visually displayed in Figure 3.

**FIGURE 4:**
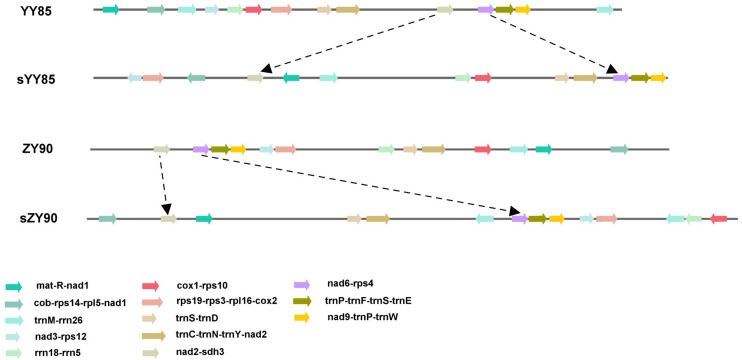
Location map of the 13 gene clusters in the four mitochondrial genomes.

The sequences of protein-coding and rRNA genes are highly conserved in the plant mitochondrial genomes, whereas the relative order of these genes varies due to frequent rearrangement. However, some highly conserved gene clusters were preserved (Bi et al., 2016). A large number of genes were close to each other, forming gene clusters that might represent co-transcriptional units. In total, 13 conserved clusters were detected in the four mitochondrial genomes (Table 3), including *mat-R-nad1*, *cob-rps14-rpl5-nad1*, *trnM-rrn26*, *nad3-rps12*, *rrn18-rrn5*, *cox1-rps10*, *rps19-rps3-rpl16-cox2*, *trnS-trnD*, *trnC-trnN-trnY-nad2*, *nad2-sdh3*, *nad6-rps4*, *trnP-trnF-trnS-trnE*, and *nad9-trnP-trnW* (Figure 4). In the *rps19-rps3-rpl16-cox2* gene cluster, *rpl16* and *rps3* had 108 bp overlapping regions in all four mitochondrial genomes. The rearrangement resulted in the two gene clusters (*nad2-sdh3* and *nad6-rps4*) tending to be close in the maintainer lines, but they were separated in the CMS lines and crossed with multiple protein-coding genes. When comparing the location of gene clusters with syntenic blocks in sYY85 and sZY90, it was found that all gene clusters were located in syntenic blocks. Among them, *rrn18-rrn5*, *cox1-rps10ab*, *nad6-rps4*, *trnP-trnF-trnS-trnE* and *nad9-trnP-trnW* in sYY85, *nad2ab-sdh3*, *nad6-rps4*, *trnP-trnF-trnS-trnE*, *nad9-trnP-trnW*, *rps12-nad3* and *rps19-rps3ab-rpl16-cox2ab* in sZY90, were located in collinearity regions of syntenic blocks. The collinearity regions were very conserved (both the sequences and the order remained the same), and the other gene clusters were located in the inversion region and translocation region of syntenic blocks. In sYY85 and sZY90, the gene cluster rearrangement was found in most of the syntenic blocks (gene cluster location and specific information are shown in Supplementary Table S3). In contrast, a previous study had reported that the small region sequences with gene clusters were protected from rearrangement events in pepper, and only *atp9-rps13-nad1bc*, *nad4-rps1-nad5ab, nad3-nad1a* and *rps4-nad6* were rearranged (Jo et al., 2014). Although the relationship of gene cluster and rearrangement was not clear, numerous rearrangements were detected in the tobacco mitochondrial genomes, and the components of these gene clusters were highly conserved. These data suggested that the genes in these conservative gene clusters were more inclined toward co-expression and co-regulation. Moreover, *rps4*-*nad6* was reported to be putative co-transcribed unit in tobacco (Sugiyama et al., 2005), and *rps3*-*rpl16*-*nad3*-*rps12* shared a common promoter and functions in a coordinated manner in rice (Nakazono et al., 1995).

**TABLE 3:** Information on the gene clusters in the four mitochondrial genomes.

Maintainer lines		CMS lines	
	
YY85 (17)	ZY90 (17)	sYY85 (17)	sZY90 (18)
*mat-R-nad1a*	*cob-D2-rps14-D2-rpl5-D2*	*rps12-nad3*	*cob-rps14-rpl5-nad1a*
*trnM-ccmC*	*nad2ab-sdh3ab*	*rps19-rps3ab-rpl16-cox2ab*	*nad2ab-sdh3*
*cob-rps14-rpl5-nad1b*	*nad6-rps4*	*nad1a-rpl5-rps14-cob*	*nad1b-rrn26-trnM*
*trnM-rrn26*(2)*	*trnP-trnF-trnS-trnE*	*orfB-cox3-atp1*	*nad1c-mat-R-D2*
*nad3-rps12*	*nad9-trnP-trnW*	*nad2ab-sdh3*	*atp9-D3-trnM*
*rrn18-rrn5*	*rps12-nad3-D2*	*nad1b-rrn26-trnM*	*rps13-nad1de*
*cox1-rps10ab*	*rps19-rps3ab-rpl16-cox2ab*	*nad1c-mat-R*	*trnS-trnD*
*nad1cd-rps13-atp9*	*ccmC-trnM*	*trnM-rrn26*	*trnC-trnN-trnY-nad2cde*
*rps19-rps3ab-rpl16-cox2ab*	*trmM-rrn26-nad1a*	*rrn18-rrn5*	*trnM-rrn26*(2)*
*trnS-trnD*	*trnS-trnD*	*atp4-nad4L*	*nad6-rps4*
*trnC-trnN-trnY-nad2abc*	*trnC-trnN-trnY-nad2cde*	*cox1-rps10ab*	*trnP-trnF-trnS-trnE*
*nad2de-sdh3*	*rps13-nad1bc*	*rps13-nad1de*	*nad9-trnP-trnW*
*nad3-D2-nad1e*	*rps10ab-cox1*	*trnS-trnD*	*rps12-nad3*
*nad6-rps4*	*rrn5-rrn18*	*trnC-trnN-trnY-nad2cde*	*rps19-rps3ab-rpl16-cox2ab*
*trnP-trnF-trnS-trnE*	*rrn26-trnM*	*nad6-rps4*	*ccmC-trnM*
*nad9-trnP-trnW*	*nad1d-mat-R*	*trnP-trnF-trnS-trnE*	*rrn18-rrn5*
	*cob-rps14-rpl5-nad1e*	*nad9-trnP-trnW*	*cox1-rps10ab*

The a, b, c, d, and e behind the gene represented exon 1, exon 2, exon 3, exon 4, and exon5, respectively. D2 represented the second copy gene, and D3 represented the third one. *Contained a copy gene cluster.

### Candidate CMS-Associated Genes in Tobacco

In tobacco, alloplasmic lines as well as protoplast fusions were generated between various species within the genus *Nicotiana* or between *Nicotiana* and other genera within the Solanaceae to obtain CMS lines for breeding and genetic studies (Burns et al., 1978; Kofer et al., 1991; Zubko et al., 2001). Rearrangement of the mitochondrial genome might produce novel chimeric ORFs. Most identified CMS-associated genes were chimeric ORFs in many crop species, fused with genes encoding ATP synthase or cytochrome c oxidase subunits (e.g., *atp6* and *cox2*) (Hanson and Bentolila, 2004; Zaegel et al., 2006). However, the CMS-associated chimeric ORFs have not yet been found in the tobacco mitochondrial genomes. The phenomenon suggested that CMS-associated genes in tobacco were different from those in other plants. In our study, specific protein-coding genes and ORFs were identified in both CMS lines of two *Nicotiana tabacum* cultivars, which were considered to be candidate CMS-associated genes in tobacco.

After aligning the protein-coding genes of both CMS lines and maintainer lines of two *Nicotiana tabacum* cultivars, it was found that 18 genes (*atp1*, *atp6*, *atp9*, *ccmB*, *ccmFN*, *cox1*, *cox2*, *cox3*, *mat-R*, *nad1*, *nad2*, *nad4*, *nad6*, *rpl5*, *rps10*, *rps13*, *rps4*, and *sdh3*) in yunyun85 showed polymorphisms with gene length and nucleotide differences, as did 6 genes (*atp6*, *ccmC*, *cox2*, *nad2*, *rps3*, and *sdh3*) in zhongyan90. Four of these genes (*atp6*, *cox2, nad2*, and *sdh3*) were common in sYY85 and sZY90, so they were considered to be the most likely candidates genes for CMS. Additional alignment of the four candidate CMS-associated genes (Table 4) showed that the lengths and sequence nucleotides of *atp6*, *nad2* and *sdh3* were conserved in the CMS lines. Synonymous and non-synonymous substitutions were detected in *atp6* of the two CMS lines. A synonymous substitution occurred in sYY85 and sZY90 at the 768th nucleotide of *atp6*. The finding suggested that there were different codon preferences in the maintainer and the CMS lines of tobacco.

**TABLE 4:** Sequence polymorphism information of candidate CMS-associated genes.

Gene	Change of length	Change of DNA sequence
*atp6*^a^	*	58 tAt (Y) → 58 tCt (S)
		91 aTg (M) → 91 aCg (T)
		253 Ttg (*) → 253 Ctg (*)
		418 Tca (S) → 418 Cca (P)
		766 tcT (*) → 766 tcC (*)
		1156 tCt (S) → 1156 tAt (Y)
*atp6*^b^	*	766 tcT (*) → 766 tcC (*)
*nad2*^a^	1467 → 1377	456–545 → – (90)^c^
		881 ggG (*) → 790 ggA (*)
*nad2*^b^	1380 → 1377	454 gcAGTt (*V) → 454 gc—t (*-)
*sdh3*^a^	327 → 312	274 acGGATTTTTCG (*DFS) → 274 ac- (10) (*—)
*sdh3*^b^	315 → 312	274 –ggat (-G) → 274 ACGgat (TD)
*cox2*^a^	*	769 aCcGGg (TG) → 769 aGcACg (ST)
*cox2*^b^	783 → 816	- (33) → 383–415^d^

^*a*^Change fromYY85 to sYY85. ^*b*^Change from ZY90 to sZY90.^*c*^Ninety bases deletion in sYY85.^*d*^Thirty-three bases insertion in sZY90.“−” Nucleotides and amino acids between the maintainer and the CMS lines were not aligned insertion or deletion.“*” Length, nucleotides and amino acids between the maintainer and the CMS lines remained unchanged. The polymorphic sites (the number indicated the position of the first nucleotide, and the polymorphic nucleotides were capitalized) and their deduced amino acids (in parentheses) from the maintainer lines were given to the left of the arrow, while those from the CMS lines were given to the right of the arrow.

A total of 248, 275, 275 and 304 ORFs (>300 bp) were predicted in YY85, sYY85, ZY90, and sZY90, respectively. After filtering out ORFs whose sequence similarities between the maintainer and the CMS lines was less than 99%, the remaining ORFs in sYY85 and sZY90 were aligned, and 16 ORFs were regarded as the specific ORFs in both CMS lines. A total of 6 CMS candidate ORFs (*orf82*, *orf103*, *orf115*, *orf91*, *orf115b*, and *orf100*) were screened and identified in sZY100 (Zheng et al., 2018), three of which (*orf91*, *orf115b*, and *orf100*) were consistent with our findings. The transcriptional analysis showed that the six ORFs were highly expressed in sZY100 and lowly expressed in ZY100. However, in our study, *orf82*, *orf103*, and *orf115* were non-specific ORFs in sYY85 and sZY90.

### CMS Specific Region of Tobacco Based on the Syntenic Analysis

**FIGURE 5:**
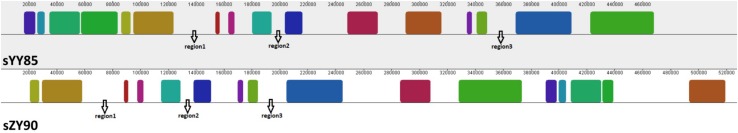
Three specific regions of the CMS mitochondrial genomes. The same color indicates the same blocks in sYY85 and sZY90.

**FIGURE 6:**
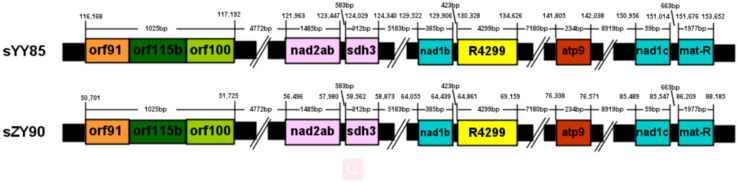
Location and information of the CMS-specific region 1.

Many CMS-associated genes were caused by rearrangements of the mitochondrial genome (Hanson and Bentolila, 2004). Recombination frequently occurred in specific regions by integrating and rearranging pre-existing mitochondrial sequences (Tanaka et al., 2012). Through comparative analysis of the YY85, sYY85, ZY90, and sZY90 mitochondrial genomes using Mauve v.2.4.0, it was found that they were comprised of 16 syntenic blocks (length > 3 kb). The CMS mitochondrial genomes had three unique regions (regions 1, 2, and 3) that were non-syntenic and distinct from the maintainer lines (Figure 5). Region 1 was the largest unique region and was close to CMS-specific ORFs, and it might be related to CMS because the similar structure was also found in pepper and radish (Tanaka et al., 2012; Jo et al., 2014). The CMS-specific region 1 of sYY85 and sZY90 (Figure 6) was 41,147 bp in length and contained five candidate CMS-associated genes, 3 protein-coding genes, 1 large repeated sequence and 52 repeated sequences distributed in the intergenic region (Supplementary Table S4). Nine small repeated sequences were located upstream and downstream of *orf91-orf115b-orf100*. Blast analysis revealed that the CMS-specific region of yunan85 and zhongyan90 also existed in zhongyan100. Therefore, this region may have important implications for tobacco CMS.

### Phylogenetic Analysis in the *Nicotiana* Genus

**FIGURE 7:**
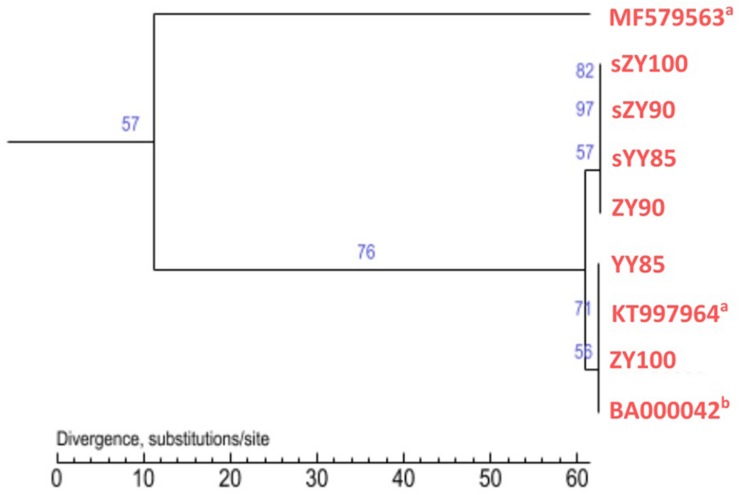
Maximum Likelihood tree based on SNPs of the tobacco mitochondrial genomes. ^a^The type of the mitochondrial genome was unknown. ^b^The type was the maintainer line.

**FIGURE 8:**
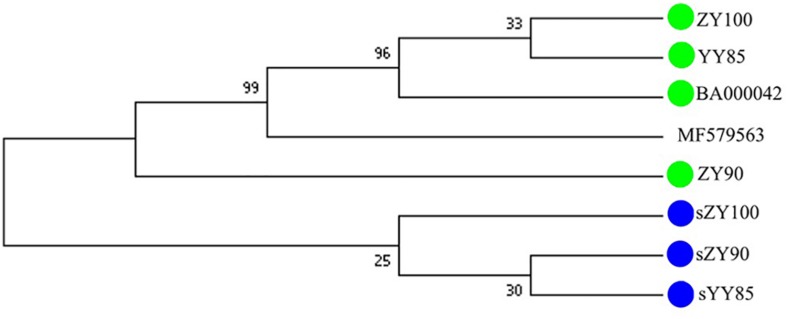
Maximum Parsimony tree based on candidate CMS-associated protein-coding genes of the tobacco mitochondrial genomes. The green and blue circles represented the maintainer and the CMS lines, respectively.

All mitochondrial genomes of the *Nicotiana* genus were collected from NCBI, including *Nicotiana tabacum*, *Nicotiana sylvestris*, and *Nicotiana attenuate* (GenBank: MF579563). Phylogenetic analyses were performed using the following two methods: Maximum Likelihood tree based on SNPs of the tobacco mitochondrial genomes (Figure 7) and Maximum Parsimony tree based on our candidate CMS-associated protein-coding genes (*atp6*, *cox2*, *nad2*, *sdh3*) (Figure 8). The tobacco maintainer lines and the CMS lines could be separated generally by the first method (except for ZY90), while these lines were separated completely by the second method. Therefore, compared to the first method, the second method could better distinguish the CMS lines from the maintainer lines. It was further suggested that *atp6*, *cox2*, *nad2* and *sdh3* might be related to CMS.

### PPI Experiment of the Candidate CMS-Associated Gene *atp6*

**FIGURE 9:**
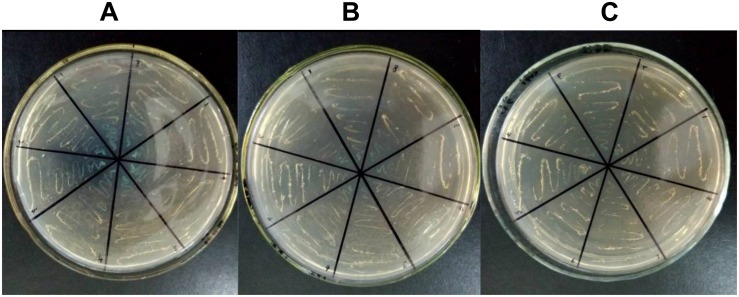
Yeast two-hybrid results of PPI for ATP6-ATP9. **(A)** Positive control; **(B)** CMS line; **(C)** maintainer line; the blue color indicated interaction.

According to previous studies, for all candidate CMS-associated genes, the relationship between *atp6* and CMS was crucial (Hanson and Bentolila, 2004; Chen and Liu, 2014). To further validate the importance of *atp6*, a PPI experiment for ATP6 was performed. Because tobacco was not included in the STRING11.0^4^ database, *Arabidopsis thaliana* was selected as the allied species for PPI prediction. The alignment results of the ATPase sequences showed that the similarity between tobacco and *Arabidopsis thaliana* was more than 70%. Therefore, it was speculated that the PPI results of *Arabidopsis thaliana* predicted in this software could better reflect the PPI tendency of tobacco. After predicting the PPI of all the ATPase proteins, the results clearly indicated that ATP9 might interact with ATP6. So ATP6 and ATP9 were chosen to verify the potential PPI relationship difference between the maintainer and the CMS lines using yeast two-hybrid technology. The results showed that the PPI for ATP6 and ATP9 in the maintainer line (Figure 9C) was weaker than that of the CMS line (Figure 9B), the blue color indicated interaction (Figure 9A). This difference in PPI intensity suggested that the *atp6* gene might be one of the key genes and important factors related to plant sterility. In future studies, the CMS-related differences will highlight *atp6* and further extend to the entire mitochondrial genome to find more key genes that may play crucial roles in sterility. A comprehensive investigation of the tobacco mitochondrial genome is helpful to improving our understanding of the sterility mechanism in Solanaceae crops.

The cytoplasm of the maintainer lines was from flue-cured tobacco cultivar, and the cytoplasm of the CMS lines was from Jiangxi sun-cured tobacco cultivar (“tiegu”). The mitochondrial genomes in our study had different cytoplasmic sources, so the differences between them are not entirely related to CMS. Understanding the differences at the mitochondrial genome level is the first step in studying the CMS mechanism. Therefore, further studies will be extended to the transcriptome and proteome to synthesize the information closely related to CMS, while carefully considering the interference caused by the different sources of maintainer cytoplasm and CMS cytoplasm.

## Conclusion

Comprehensive analyses of the mitochondrial genomes of YY85, sYY85, ZY90, and sZY90 demonstrated that extensive structural variations, including rearrangement, gene order, genomic expansion and shrinkage events, might be related to CMS. More specifically, four candidate CMS-specific genes (*atp6*, *nad2*, *cox2*, and *sdh3*) and the 16 candidate CMS-specific ORFs were closely associated with the CMS mechanism. They not only represented candidate CMS-associated genes, but they could also be used as molecular markers to identify the CMS lines in tobacco. Notably, the PPI experiment for *atp6* further supported the importance of this candidate CMS-specific gene and the validity of our research results. An independent analysis of the mitochondrial genome information is not sufficient to reveal the CMS mechanism. Therefore, more valuable information from multi-omics research is urgently needed to carry out the follow-up work.

## Data Availability Statement

The datasets generated for this study can be found in the GenBank: MN651321, MN651322, MN651323, and MN651324.

## Author Contributions

WZ and QL designed the research and revised manuscript. RW conducted the research and drafted the manuscript. SH and YL conducted the molecular work and data analysis. XC and YF and prepared the figures and tables. ST checked the manuscript. All authors reviewed and approved the manuscript.

## Conflict of Interest

The authors declare that the research was conducted in the absence of any commercial or financial relationships that could be construed as a potential conflict of interest.

## Footnotes

1 https://blast.ncbi.nlm.nih.gov/Blast.cgi

2 http://www.repeatmasker.org/cgi-bin/WEBRepeatMasker

3 http://bioinf.uni-greifswald.de/augustus/

4 https://www.string-db.org/
